# Association of Abdominal Aortic Calcification with Peripheral Quantitative Computed Tomography Bone Measures in Older Women: The Perth Longitudinal Study of Ageing Women

**DOI:** 10.1007/s00223-022-01016-5

**Published:** 2022-08-13

**Authors:** Jack Dalla Via, Marc Sim, John T. Schousboe, Douglas P. Kiel, Kun Zhu, Jonathan M. Hodgson, Abadi K. Gebre, Robin M. Daly, Richard L. Prince, Joshua R. Lewis

**Affiliations:** 1 Nutrition and Health Innovation Research Institute, School of Medical and Health Sciences, Edith Cowan University, Perth, WA Australia; 2 Medical School, The University of Western Australia, Perth, WA Australia; 3 Park Nicollet Osteoporosis Center and Health Partners Institute, Minneapolis, MN USA; 4 Division of Health Policy and Management, University of Minnesota, Minneapolis, MN USA; 5 Hinda and Arthur Marcus Institute for Aging Research, Hebrew SeniorLife, Department of Medicine Beth Israel Deaconess Medical Center and Harvard Medical School, Boston, MA USA; 6 Department of Endocrinology and Diabetes, Sir Charles Gairdner Hospital, Perth, WA Australia; 7 School of Pharmacy, College of Health Sciences, Mekelle University, Mekelle, Tigray, Ethiopia; 8 Institute for Physical Activity and Nutrition, School of Exercise and Nutrition Sciences, Deakin University, Geelong, VIC Australia; 9 Centre for Kidney Research, Children’s Hospital at Westmead School of Public Health, Sydney Medical School, The University of Sydney, Sydney, NSW Australia

**Keywords:** Abdominal aortic calcification, Vascular calcification, Bone, Peripheral quantitative computed tomography, Older women

## Abstract

We have previously shown that abdominal aortic calcification (AAC), a marker of advanced atherosclerotic disease, is weakly associated with reduced hip areal bone mineral density (aBMD). To better understand the vascular–bone health relationship, we explored this association with other key determinants of whole-bone strength and fracture risk at peripheral skeletal sites. This study examined associations of AAC with peripheral quantitative computed tomography (pQCT)-assessed total, cortical and trabecular volumetric BMD (vBMD), bone structure and strength of the radius and tibia among 648 community-dwelling older women (mean ± SD age 79.7 ± 2.5 years). We assessed associations between cross-sectional (2003) and longitudinal (progression from 1998/1999–2003) AAC assessed on lateral dual-energy X-ray absorptiometry (DXA) images with cross-sectional (2003) and longitudinal (change from 2003 to 2005) pQCT bone measures at the 4% radius and tibia, and 15% radius. Partial Spearman correlations (adjusted for age, BMI, calcium treatment) revealed no cross-sectional associations between AAC and any pQCT bone measures. AAC progression was not associated with any bone measure after adjusting for multiple comparisons, despite trends for inverse correlations with total bone area at the 4% radius (r_s_ =  − 0.088, *p* = 0.044), 4% tibia (r_s_ =  − 0.085, *p* = 0.052) and 15% radius (r_s_ =  − 0.101, *p* = 0.059). Neither AAC in 2003 nor AAC progression were associated with subsequent 2-year pQCT bone changes. ANCOVA showed no differences in bone measures between women with and without AAC or AAC progression, nor across categories of AAC extent. Collectively, these finding suggest that peripheral bone density and structure, or its changes with age, are not associated with central vascular calcification in older women.

**Supplementary Information:**

The online version contains supplementary material available at 10.1007/s00223-022-01016-5.

## Introduction

Bone and vascular diseases have been shown to be biologically related. For example, vascular calcification is thought to be due to perturbations of pro- or anti-calcific signalling molecules, and shares common pathophysiological mechanisms with bone loss [1, 2]. During ageing, bone mass is progressively lost while blood vessels accumulate pathological mineralised deposits, suggesting that vascular disease may compromise blood flow to the skeleton. Indeed, cardiovascular disease has been shown to be associated with osteoporosis and future fracture risk [3, 4].

Of particular interest is abdominal aortic calcification (AAC), a marker of advanced vascular disease and generalised atherosclerosis [5]. Presence of AAC is reported to be predictive of cardiovascular events and cardiovascular-related mortality [6]. AAC can be assessed on images from abdominal and lumbar computed tomography, standard lateral spine thoracolumbar radiographs or thoracolumbar lateral spine images from dual-energy X-ray absorptiometry (DXA), which is typically used to assess bone mineral density (BMD).

Previously, we have shown AAC is inversely associated with areal BMD (aBMD) of the hip and broadband ultrasound attenuation of the calcaneus in older women, as well as long-term risk of being hospitalised with a fracture [7]. However, DXA is a two-dimensional imaging modality that cannot assess important factors contributing to bone strength beyond the mineral content of a small region of interest in a given location within a bone. Alternatively, peripheral quantitative computed tomography (pQCT) is able to assess three-dimensional measures at various skeletal sites, including volumetric BMD (vBMD), and bone geometric properties, such as bone size and shape, distinguish cortical and trabecular components and provide estimates of bone strength [8]. This may be of particular importance given the observation that individuals with type 2 diabetes, who often have significant atherosclerotic vascular disease, may have differential effects on cortical and trabecular bone [9]. Bone structural measures obtained via high-resolution (HR) pQCT have been shown to be predictive of fracture risk in older adults, independent of DXA-assessed bone density [10]. Women, particularly those who are past menopause, have been shown to have a greater rate of cortical and trabecular bone loss, as well as more rapid impairments in bone structure and strength compared to men, contributing to their higher risk of fracture [11, 12]. Therefore, investigating associations between AAC and cortical and trabecular bone structure is warranted to build on the previously reported association with hip aBMD, and develop a more comprehensive understanding of the relationship between vascular disease and bone health.

To date, no study has investigated the relationship between AAC from single-energy lateral spine images from DXA machines and pQCT bone measures. The measurement of peripheral appendicular sites with three-dimensional bone measures using pQCT may provide further insights into the relationship between vascular calcification and bone loss. Therefore, the aim of this study was to investigate AAC presence, extent and progression with total, cortical and trabecular volumetric bone density, bone geometry and strength and its changes over time among older women. It is hypothesised that women with more extensive AAC will have poorer cortical and trabecular vBMD, bone structure and bone strength.

## Methods

### Study Population

Participants included in the current secondary analysis represent a subsample of women participating in the Perth Longitudinal Study of Ageing in Women (PLSAW). These women were originally enrolled in a 5-year randomised controlled trial of oral calcium supplementation and osteoporotic fractures (Calcium Intake Fracture Outcome Study, CAIFOS) [13], which continued as the PLSAW. For the present study, women from the PLSAW who had valid AAC results and pQCT scans at the end of the RCT in 2003 were included. Participants were excluded if they were missing covariate data or if they were treated with bisphosphonates or warfarin prior to their 2003 clinic visit given their effects on bone and/or vascular calcification. All participants provided written informed consent. Ethics approval was granted by the Human Ethics Committee of the University of Western Australia. Both studies were retrospectively registered on the Australian New Zealand Clinical Trials Registry (CAIFOS trial registration number #ACTRN12615000750583 and PLSAW trial registration number #ACTRN12617000640303) and complied with the Declaration of Helsinki.

### Baseline Characteristic Assessment

For this study we considered the baseline to be measurements in 2003, as this was the year of the first pQCT measurements available. Body weight was measured using digital scales and height was assessed using a wall-mounted stadiometer, with participants wearing light clothing and no shoes. Body mass index (BMI) was then calculated in kg/m^2^. Treatment allocation (placebo or calcium) from the CAIFOS trial was included as a covariate. Smoking status was categorised as either non-smoker or smoked ever (currently or previously a smoker). Prevalent atherosclerotic vascular disease (ASVD) was determined using primary discharge diagnoses from hospital records, as described previously [14]. Diabetes status was determined by the use of medications for diabetes (oral hypoglycaemic agents or insulin). Physical activity was assessed via questionnaire, where participants reported the frequency and duration of sports recreation or regular physical activity in the previous three months, as reported in detail previously for this cohort [15]. This information, along with the energy cost of the activity and body weight of the participant, was used to calculate activity levels in kcal/day.

### Abdominal Aortic Calcification

AAC was assessed from lateral spine DXA scans, obtained at the baseline for this study (2003) and 4–5 years prior (1998/99) using a Hologic 4500A scanner (Hologic, Marlborough, MA, USA). AAC was scored from 0 to 24 (AAC24) using established methods [16–18] by a single blinded experienced investigator (JTS). Presence of AAC was categorised as either having (AAC24 score ≥ 1) or not having (AAC24 score of 0) evidence of AAC at the baseline bone structure evaluation for this analysis. AAC was categorised using established [16, 19] groupings of the extent of AAC for CVD outcomes: low (AAC24 score 0 or 1), moderate (AAC24 score 2–5) and extensive (AAC24 score ≥ 6). Similarly, progression of AAC in the 4–5 years prior to baseline was categorised as either no progression (increase in AAC24 ≤ 1) or progression (increase in AAC24 > 1). AAC24 assessment is shown to be reproducible over a 4-year period [20], with very high intra-rater reliability reported from DXA-derived images (intraclass correlation coefficient: 0.91–0.95) [21]. Importantly, the same highly experienced trained imaging specialist (JTS) read both the baseline and the previous image together to determine the change, blinded to clinical data. Nevertheless, an increase in AAC24 score of one was considered as no progression to minimise the risk of misclassification due to possible image quality issues.

### pQCT Bone Measurements

pQCT scans of the 4% site of the distal tibia and both the 4% and 15% sites of the distal radius were obtained at the baseline visit of this analysis (2003) and two years later (2005) using a Stratec XCT 2000 scanner (Stratec Medizintechnik GmbH, Pforzheim, Germany). The 4% site for the radius and tibia predominantly consists of trabecular bone, while the 15% site contains more cortical bone than trabecular bone. The slice thickness was 1 mm and voxel size was 0.15 mm. pQCT images were analysed using the Stratec software. Thresholds of 169 and 710 mg/cm^3^ were used to define total and cortical bone, respectively. Parameters measured at the 4% sites included total and trabecular bone mineral content (mg/mm), volumetric density (vBMD; mg/cm^3^), bone area (mm^2^) and estimated compressive bone strength (BSI_C_; mg^2^/mm^4^). BSI_C_ was calculated as total bone cross-sectional area multiplied by the square of total bone density [22]. Parameters measured at the 15% radius included total, and cortical and subcortical bone mineral content (mg/mm), volumetric density (vBMD; mg/cm^3^) and bone area (mm^2^), as well as the polar section modulus (mm^3^), polar moment of inertia (mm^4^) and stress–strain index (mm^3^) [23, 24]. Stress–strain index of cortical bone was calculated as the product of the section modulus and cortical density normalised to the maximal physiological cortical density of human bones (1200 mg/cm^3^) for the polar moment (SSI_polar_) and the bending moments in the x (SSI_x_) and y (SSI_y_) axes, where the y axis is the widest part of the tibia and the x axis is perpendicular to this [23, 24]. The CVs for total, cortical and subcortical, and trabecular vBMD were 4.6%, 8.0%, and 4.0%, respectively. Bone measures were analysed cross-sectionally in all participants, and longitudinally (2-year change from baseline) in participants with valid pQCT scans at both time points. The reference line for the 2-year follow-up scans was registered at the same place as baseline. Bone change data were checked for outliers, with percentage changes greater than three standard deviations either side of the mean percentage change for each measure classified as an outlier and excluded from the longitudinal bone analyses (*n* = 4–9, depending on bone site and outcome).

### Statistical Analysis

Participant characteristic data are presented as mean ± SD, median and interquartile range (IQR), or number and percent as appropriate. Partial Spearman’s rank-order correlation analyses, adjusted for age, BMI and the original treatment allocation (calcium/placebo), were used to assess associations between AAC and bone measures. Spearman rank-order analysis was used as AAC data (both AAC24 scores at baseline, and AAC progression) were positively skewed due to a large proportion of women with no AAC or progression. Analyses of AAC progression were additionally adjusted for initial AAC24 scores. Analyses of longitudinal bone measures were adjusted for baseline bone data. Bone measures were also compared between women with and without AAC (AAC24 score of 0 vs ≥ 1) and across categories of AAC extent (low [AAC24 = 0–1], moderate [AAC24 = 2–5], extensive [AAC24 ≥ 6]) using ANCOVA, adjusted for age, BMI and the original treatment allocation. Comparisons between women with and without AAC progression (AAC24 increase ≤ 1 vs. increase in AAC24 > 1) were additionally adjusted for AAC24 scores 4–5 years prior to baseline. Statistical analyses were performed using IBM SPSS Statistics 27 (IBM Corp., Armonk, NY, USA). To adjust for multiple comparisons, a significance level of *p* < 0.001 was adopted for all correlation analyses. A significance level of *p* < 0.05 was adopted for Bonferroni-adjusted ANCOVA analyses.

### Additional Analysis

Sensitivity analyses were conducted with physical activity and smoking status included as additional covariates, with women treated with bisphosphonates and/or warfarin included in this analysis, with women with type 2 diabetes excluded from this analysis and without adjustment for baseline AAC and/or baseline bone data (for longitudinal analyses).

## Results

### Participant Characteristics

**Fig. 1:**
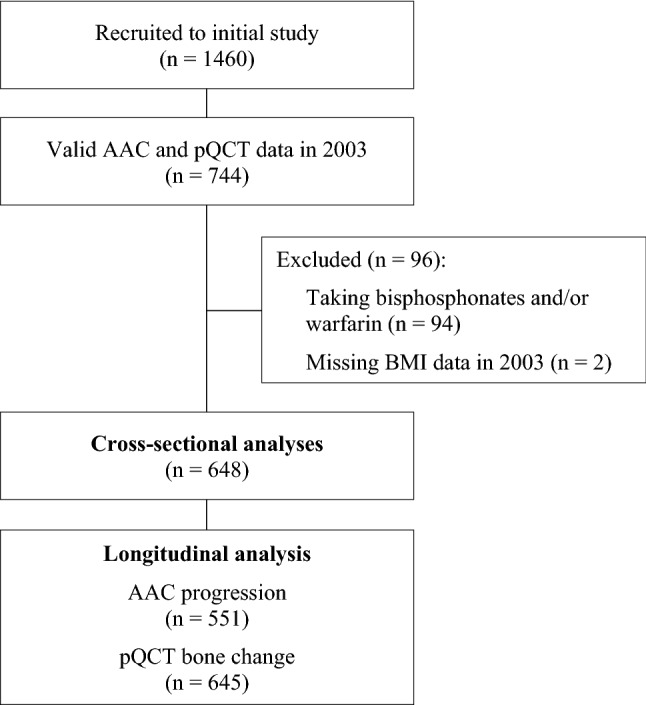
Participant flowchart showing how the study population was derived. For pQCT, participants were included if they had data available for at least one of the three scan sites. Therefore, the number of participants specified here for the longitudinal analysis differs from the number of participants actually included in the analysis for each individual bone measure

A total of 648 women were included in the current study, with a mean (SD) age of 79.7 (2.5) years at the baseline for this analysis. While 744 women had valid AAC and pQCT data at baseline, 94 women were excluded for bisphosphonate and/or warfarin use (*n* = 54 for bisphosphonates; *n* = 36 for warfarin; *n* = 4 for both), and another two women were excluded due to missing BMI data (Fig. 1). For longitudinal analyses, 551 women had valid data on AAC progression, while 645 women had valid pQCT bone change data from at least one bone site. There were no differences in demographic, AAC or pQCT bone measures between women with and without valid longitudinal AAC or bone data (data not shown). Characteristics of the final sample at baseline are presented in Table 1.

**Table 1:** Participant characteristics at the baseline for this analysis (2003)

Demographics	All participants
Number	648
Age, years	79.7 ± 2.5
Height, cm	158 ± 5.7
Weight, kg	67.6 ± 11.8
BMI, kg/m^2^	27.2 ± 4.5
Ever a smoker^a^, n (%)	224 (34.7%)
*Treatment allocation*	
Calcium, n (%)	319 (49.2%)
Placebo, n (%)	329 (50.8%)
Prevalent ASVD, n (%)	87 (13.4%)
Physical activity^b^, kcal/day	118 (45, 199)
AAC present (AAC24 ≥ 1), n (%)	517 (79.8%)
*AAC extent*	
Low (AAC24 = 0–1), n (%)	224 (34.6%)
Moderate (AAC24 = 2–5), n (%)	259 (40.0%)
Extensive (AAC24 ≥ 6), n (%)	165 (25.5%)
*pQCT bone measures*	
*4% Radius*^*c*^	
Total bone content (mg/mm)	82.1 ± 16.5
Total bone area (mm^2^)	282 ± 54
Total bone density (mg/cm^3^)	298.4 ± 68.5
Trabecular bone content (mg/mm)	40.3 ± 11.8
Trabecular bone area (mm^2^)	218 ± 48
Trabecular bone density (mg/cm^3^)	188 ± 49
BSI_C_ (g^2^/cm^4^)	0.252 ± 0.099
*4% Tibia*^*d*^	
Total bone content (mg/mm)	281 ± 49
Total bone area (mm^2^)	1192 ± 132
Total bone density (mg/cm^3^)	237 ± 43
Trabecular bone content (mg/mm)	231 ± 46
Trabecular bone area (mm^2^)	1070 ± 127
Trabecular bone density (mg/cm^3^)	218 ± 44
BSI_C_ (g^2^/cm^4^)	0.684 ± 0.228
*15% Radius*^*e*^	
Total bone content (mg/mm)	74.6 ± 13.3
Total bone area (mm^2^)	117 ± 19
Total bone density (mg/cm^3^)	646 ± 111
Cortical and subcortical bone content (mg/mm)	64.3 ± 12.5
Cortical and subcortical bone area (mm^2^)	56.0 ± 10.3
Cortical and subcortical bone density (mg/cm^3^)	1149 ± 87
Polar section modulus (mm^3^)	78.6 ± 31.2
Polar moment of inertia (mm^4^)	643 ± 276
SSI_polar_ (mm^3^)	219 ± 49
SSI_x_ (mm^3^)	109 ± 23
SSI_y_ (mm^3^)	148 ± 35

Data are mean ± SD, number n (%), or median (interquartile range). ^a^ *n* = 646, ^b^ *n* = 641, ^c^ *n* = 622, ^d^ *n* = 629, ^e^ *n* = 409. BMI = Body Mass Index, ASVD = Atherosclerotic Vascular Disease, AAC = Abdominal Aortic Calcification, BSI_C_ = Compressive Bone Strength Index, SSI = Stress–Strain Index

### AAC and Cross-Sectional Bone Measures

AAC was present in 79.8% of women at baseline, with 34.6%, 40.0% and 25.5% classified as having low, moderate and extensive AAC, respectively (Table 1). AAC progression in the 4–5 years prior to baseline was observed in 29.6% of women. The mean increase in AAC24 score among women with AAC progression was 3.3 (95%CI 3.1, 3.6).

At baseline, there were no significant cross-sectional associations between AAC24 score and any bone measure (Table 2). After adjusting for multiple comparisons, progression of AAC in the 4–5 years prior to baseline was also not associated with any bone measure (Table 2), despite trends for correlations with a smaller total bone area at the 4% radius (r_s_ =  − 0.088, *p* = 0.044), 4% tibia (r_s_ =  − 0.085, *p* = 0.052) and 15% radius (r_s_ =  − 0.101, *p* = 0.059). None of the bone measures at any skeletal site differed between women with or without AAC present at baseline, with or without AAC progression in the 4–5 years prior to baseline, nor across categories of AAC extent at baseline (Supplementary Tables 1–3).

**Table 2:** Associations between abdominal aortic calcification (AAC) at baseline or AAC progression in the 4–5 years prior to baseline, and baseline bone measures at the distal 4% radius and tibia, and 15% radius

	AAC24 at baseline		AAC progression in the 4–5 years prior to baseline	
	r_s_	*p* value	r_s_	*p* value
*4% Radius*	*n* = *622*		*n* = *530*	
Total bone content (mg/mm)	− 0.019	0.630	− 0.014	0.751
Total bone area (mm^2^)	− 0.013	0.738	− 0.088	0.044
Total bone density (mg/cm^3^)	− 0.006	0.884	0.037	0.392
Trabecular bone content (mg/mm)	0.006	0.883	0.015	0.732
Trabecular bone area (mm^2^)	0.001	0.971	− 0.082	0.061
Trabecular bone density (mg/cm^3^)	− 0.003	0.948	0.068	0.119
BSI_C_ (g^2^/cm^4^)	− 0.013	0.756	0.020	0.644
*4% Tibia*	*n* = *629*		*n* = *534*	
Total bone content (mg/mm)	0.009	0.814	0.026	0.557
Total bone area (mm^2^)	− 0.047	0.243	− 0.085	0.052
Total bone density (mg/cm^3^)	0.037	0.351	0.080	0.067
Trabecular bone content (mg/mm)	0.014	0.728	0.028	0.515
Trabecular bone area (mm^2^)	− 0.041	0.302	− 0.079	0.068
Trabecular bone density (mg/cm^3^)	0.035	0.381	0.075	0.083
BSI_C_ (g^2^/cm^4^)	0.028	0.482	0.061	0.162
*15% Radius*	*n* = *409*		*n* = *351*	
Total bone content (mg/mm)	0.013	0.794	− 0.067	0.210
Total bone area (mm^2^)	0.021	0.670	− 0.101	0.059
Total bone density (mg/cm^3^)	− 0.003	0.955	0.009	0.866
Cortical and subcortical bone content (mg/mm)	0.000	0.992	− 0.050	0.358
Cortical and subcortical bone area (mm^2^)	− 0.039	0.433	− 0.097	0.072
Cortical and subcortical bone density (mg/cm^3^)	0.043	0.387	0.084	0.120
Polar section modulus (mm^3^)	0.014	0.780	0.008	0.883
Polar moment of inertia (mm^4^)	0.010	0.843	− 0.006	0.915
SSI_polar_ (mm^3^)	0.050	0.314	− 0.080	0.137
SSI_x_ (mm^3^)	0.030	0.548	− 0.079	0.143
SSI_y_ (mm^3^)	0.036	0.469	− 0.062	0.247

r_s_ = Spearman rank-order correlations, adjusted for age, BMI and original treatment allocation. Analyses of AAC progression additionally adjusted for initial AAC24. BSI_C_ = Compressive Bone Strength Index, SSI = Stress–Strain Index

### AAC and Longitudinal Bone Change

Total vBMD and estimated bone strength at each bone site, as well as cortical and subcortical vBMD at the 15% radius decreased in the 2 years following baseline (mean changes –0.4% to –3.8%). Trabecular vBMD and total bone area did not change at the 4% radius, but declined at the 4% tibia (mean changes –0.4% to –1.2%). Total bone area increased at the 15% radius (mean change 2.4%, 95%CI 1.8, 3.0), but there was no change in cortical and subcortical area.

Neither AAC24 score at baseline nor AAC progression in the 4–5 years prior to baseline was associated with subsequent 2-year changes in total, trabecular, or cortical and subcortical bone vBMD, cross-sectional area or estimated strength at any bone site (Table 3). Women with AAC present at the baseline visit for this analysis had a greater decline in cortical and subcortical bone density at the 15% radius (− 3.1% vs. − 1.2%, *p* = 0.042) (Supplementary Table 1), but there were no other differences in longitudinal bone changes at any site between women with or without AAC present, with or without AAC progression, or across categories of AAC extent (Supplementary Tables 1–3).

**Table 3:** Associations between abdominal aortic calcification (AAC) at baseline or AAC progression in the 4–5 years prior to baseline, and longitudinal bone measures (change in 2 years following baseline) at the distal 4% radius and tibia, and 15% radius

	**AAC24 at baseline**			**AAC progression in the 4–5 years prior to baseline**		
	***n***	**r**_**s**_	***p* value**	***n***	**r**_**s**_	***p* value**
*4% Radius*						
∆ Total bone area (mm^2^)	540	0.002	0.970	462	–0.019	0.680
∆ Total bone density (mg/cm^3^)	542	–0.029	0.509	462	0.066	0.156
∆ Trabecular bone area (mm^2^)	540	0.002	0.956	462	–0.021	0.658
∆ Trabecular bone density (mg/cm^3^)	540	0.063	0.145	461	0.056	0.230
∆ BSI_C_ (g^2^/cm^4^)	544	–0.019	0.654	464	0.082	0.078
*4% Tibia*						
∆ Total bone area (mm^2^)	597	0.033	0.422	510	0.034	0.452
∆ Total bone density (mg/cm^3^)	597	0.010	0.811	508	0.041	0.365
∆ Trabecular bone area (mm^2^)	596	0.011	0.794	509	0.006	0.891
∆ Trabecular bone density (mg/cm^3^)	599	–0.018	0.668	510	0.017	0.700
∆ BSI_C_ (g^2^/cm^4^)	598	0.022	0.597	509	0.048	0.279
*15% Radius*						
∆ Total bone area (mm^2^)	368	0.070	0.181	319	0.047	0.409
∆ Total bone density (mg/cm^3^)	369	–0.069	0.188	319	–0.004	0.941
∆ Cortical and subcortical bone area (mm^2^)	368	0.024	0.642	318	0.024	0.678
∆ Cortical and subcortical bone density (mg/cm^3^)	369	–0.101	0.054	319	–0.084	0.138
∆ SSI_polar_ (mm^3^)	366	–0.051	0.332	316	–0.035	0.539

r_s_ = Spearman rank-order correlations, adjusted for age, BMI, original treatment allocation and 5-year bone data. Analyses of AAC progression additionally adjusted for initial AAC24. BSI_C_ = Compressive Bone Strength Index, SSI = Stress–Strain Index

### Additional Analysis

There were no meaningful differences in the results for any analyses when physical activity and smoking status were included as additional covariates. Similarly, results were consistent when women treated with bisphosphonates and/or warfarin were included in this analysis, and when 43 women with type 2 diabetes were excluded from this analysis. Results were consistent with and without adjusting longitudinal analyses for baseline AAC and/or baseline bone data.

## Discussion

The main findings from this study were that neither AAC scores nor the progression of AAC were associated with pQCT-derived radius or tibia total, cortical or trabecular bone density, structure or strength measures, nor their changes over two years, in community-dwelling older women. There were also no consistent differences in pQCT bone measures between women with and without AAC or AAC progression, nor across categories of AAC extent.

These data are in contrast with DXA-derived bone data we have previously reported among 1024 women from same cohort in which cross-sectional AAC24 scores were weakly inversely associated with hip aBMD (r_s_ =  − 0.075, *p* = 0.017) [7]. This suggests that the relationship with AAC differs between more central and peripheral skeletal sites. The relationship between AAC and hip aBMD in our cohort is supported by cross-sectional studies in healthy middle-aged and older women reporting that arterial calcification is inversely associated with QCT-assessed vBMD of the lumbar spine [25–27]. Arterial calcification is also shown to be inversely associated with vertebral trabecular vBMD in various clinical populations [28–30]. Our negative results are, however, consistent with some previous cross-sectional studies in women that report no association between vertebral trabecular vBMD and CT-assessed AAC in multivariable-adjusted analyses [31–33]. However, these comparisons are limited by most studies measuring AAC quantitatively from CT scans, as opposed to semi-quantitatively from lateral DXA scans in the current study. Collectively, the available evidence tends to suggest that arterial calcification may be inversely associated with vertebral and hip BMD, but not tibia or radius outcomes, in community-dwelling women. It is possible that AAC is more likely to be associated with bone measures at skeletal sites closer to the abdominal aorta as opposed to the peripheral skeletal sites assessed in the current study. These inconsistencies may also relate to central and peripheral bone structures being only weakly related [34]. However, there are various shared genetic and environmental risk factors that are systemically related to both vascular calcification and low BMD, which would suggest a consistent relationship between vascular calcification and bone throughout the skeleton [35]. Furthermore, the comparatively lower sample size in the current analysis, as well as potentially greater variation in pQCT compared to DXA-derived bone data, may have contributed to the lack of observed associations.

It is possible that calcification in peripheral arteries is more closely associated with bone measures at peripheral skeletal sites. [36–38]. Older women with lower leg arterial calcification were shown to have impaired HR-pQCT-assessed bone microarchitecture, but not bone area or vBMD, at the 4% distal tibia compared to women without arterial calcification [36]. Similarly, lower leg arterial calcification has been shown to be negatively associated with total, cortical and trabecular vBMD at the tibia and radius in Gambian women [37], and with poorer bone microarchitecture at the distal tibia in chronic kidney disease patients [38]. Overall, emerging evidence suggests that bone outcomes in the peripheral skeleton may relate to calcification in peripheral but not central vasculature.

We did not observe any differences in the relationship between AAC and bone outcomes at the distal tibia compared to radius. Terminal branches of the abdominal aorta supply blood to the lower limbs, so it may have been expected that AAC would be more strongly associated with bone measures at the tibia compared to radius. While no other study assessing AAC and bone has measured outcomes at both peripheral skeletal sites, previous studies have reported similar associations between lower leg arterial calcification and bone outcomes at the tibia and radius [36, 38]. We also showed that associations with AAC were equivalent between cortical and trabecular bone measures at the distal radius. This is consistent with studies in older women reporting no association between AAC and both trabecular and cortical vBMD at the femoral neck [31, 33], but are in contrast with studies reporting stronger inverse correlations between AAC or peripheral calcification with trabecular compared to cortical bone [25, 29, 37]. Decreased blood flow to the trabecular compartment due to atherosclerosis has been hypothesised as a possible reason why arterial calcification may be more strongly associated with trabecular bone [25, 37]. This is supported by evidence that decreased bone marrow perfusion is associated with lower vertebral BMD [39]. However, type 2 diabetics, who often have significant atherosclerotic vascular disease, have been shown to have impaired cortical bone density and microarchitecture at the tibia compared to non-diabetics, with no differences in trabecular outcomes [9]. Overall, it remains unclear whether the relationship with arterial calcification differs between peripheral bone sites, or between cortical and trabecular bone.

Previous studies investigating the relationship between AAC and three-dimensional bone measures have almost exclusively been cross-sectional. Our study is among the first to consider longitudinal progression of AAC and change in vBMD, bone structure and strength. Our results suggest that neither AAC nor the progression of AAC in the preceding years is associated with subsequent change in bone measures over the following two years. In contrast, a previous longitudinal analysis including 228 healthy postmenopausal women reported that greater annual gains in aortic calcification was associated with greater annual declines in CT-derived vertebral trabecular vBMD [27]. Similarly, progression of AAC in older women has been shown to be associated with greater metacarpal bone loss, as assessed by manual measurement of hand radiographs [40, 41]. However, inconsistency in the assessment of AAC and/or bone in these previous longitudinal studies limit comparison to the current results.

This study has several strengths. Firstly, the inclusion of longitudinal data allowed for analyses, including both progression of AAC over 4–5 years and change in bone measures over 2 years, which is novel in the literature. This is also the first known study to assess bone parameters at a long bone diaphysis (i.e. 15% radius). Furthermore, we included measures of bone structure and estimated bone strength in addition to total, cortical and trabecular bone density. Additionally, AAC was scored by a highly experienced investigator (JTS) who assessed all lumbar spine images. Nevertheless, a number of limitations must be acknowledged. Firstly, this was an observational study; thus, causality cannot be determined. Secondly, this study only included community-dwelling older women, so results may not be generalisable to men or to clinical populations. Thirdly, only a subsample of women from the original cohort completed pQCT scans, potentially limiting statistical power of the current analysis and introducing survival bias. Data for AAC progression and change in bone measures only included women with valid data at both time points, which further reduced the sample size for longitudinal analyses. Moreover, the precision of estimated changes of AAC on densitometric lateral spine images is unknown, and if semi-quantitative scored changes of AAC are imprecise that would limit our ability to capture associations of AAC changes with other measures. Additionally, longitudinal pQCT data were only analysed over a 2-year time period, which may be insufficient considering the expected annual changes in these bone measures are relatively small in comparison to the measurement variability with pQCT [42]. Compared to pQCT used in this study, HR-pQCT would also have provided more in depth bone microarchitecture measures at distal peripheral sites. Furthermore, we were unable to assess cortical bone measures of the tibia as data for the 15% site of the tibia were not available at baseline. A site closer to the mid-diaphysis of the radius (often 50% or 66%) is also more commonly used to assess cortical bone using pQCT. Finally, given people with more extensive AAC are less likely to survive, we cannot exclude the possibility of survivor bias leading to a type II error when investigating the association of change in AAC with bone outcomes.

In conclusion, the findings from this study indicate that AAC is not associated with total, cortical or trabecular vBMD, nor with bone structure or estimated bone strength at the tibia or radius in older women, nor the longitudinal change in these measures. These findings suggest that peripheral bone density and structure, or its changes with age, are not associated with central vascular calcification in older women. Further research is required to better understand the relationship between vascular calcification and important determinants of whole-bone strength, beyond simply bone mineral density.

## Supplementary Information

Below is the link to the electronic supplementary material.

Supplementary file1 (DOCX 74 KB)
