# Supplementary material for: Association of Abdominal Aortic Calcification with Peripheral Quantitative Computed Tomography Bone Measures in Older Women: The Perth Longitudinal Study of Ageing Women
Source: Calcif Tissue Int. 2022 Aug 13;111(5):485–94. doi: 10.1007/s00223-022-01016-5 (PMC9560937; doi:10.1007/s00223-022-01016-5)
Supplement: Supplementary file 1 — Supplementary file1 (DOCX 74 KB) [file 223_2022_1016_MOESM1_ESM.docx]

**Supplementary Table 1.** Cross-sectional and longitudinal bone measures stratified by presence of abdominal aortic calcification (AAC) at baseline.

|  | | **Presence of AAC at baseline** | | | | | | |
| --- | --- | --- | --- | --- | --- | --- | --- | --- |
|  | | **Yes (AAC24 ≥ 1)** | | | **No (AAC24 = 0)** | | ***p* value** | |
| **Cross-sectional bone measures (baseline)** | |  | | |  | |  | |
| *4% Radius* | | *n=495* | | | *n=127* | |  | |
| Total bone content (mg/mm) | | 81.6 (80.3, 82.9) | | | 83.9 (81.4, 86.5) | | 0.113 | |
| Total bone area (mm^2^) | | 281 (277, 286) | | | 282 (272, 291) | | 0.901 | |
| Total bone density (mg/cm­^3^) | | 297 (291, 303) | | | 304 (293, 316) | | 0.238 | |
| Trabecular content (mg/mm) | | 40.2 (39.2, 41.2) | | | 40.6 (38.6, 42.6) | | 0.726 | |
| Trabecular area (mm^2^) | | 218 (213, 222) | | | 217 (209, 226) | | 0.963 | |
| Trabecular density (mg/cm­^3^) | | 188 (184, 192) | | | 190 (182, 198) | | 0.627 | |
| BSI­_C_ (g^2^/cm^4^) | | 0.249 (0.241, 0.257) | | | 0.264 (0.248, 0.280) | | 0.085 | |
| *4% Tibia* | | *n=501* | | | *n=128* | |  | |
| Total bone content (mg/mm) | | 281 (278, 285) | | | 278 (271, 286) | | 0.495 | |
| Total bone area (mm^2^) | | 1189 (1177, 1200) | | | 1203 (1181, 1226) | | 0.259 | |
| Total bone density (mg/cm­^3^) | | 239 (235, 242) | | | 233 (226, 240) | | 0.152 | |
| Trabecular bone content (mg/mm) | | 232 (228, 236) | | | 229 (222, 236) | | 0.466 | |
| Trabecular bone area (mm^2^) | | 1067 (1056, 1078) | | | 1080 (1058, 1102) | | 0.326 | |
| Trabecular bone density (mg/cm­^3^) | | 219 (216, 223) | | | 213 (206, 221) | | 0.156 | |
| BSI­_C_ (g^2^/cm^4^) | | 0.688 (0.670, 0.707) | | | 0.666 (0.630, 0.702) | | 0.283 | |
| *15% Radius* | | *n=325* | | | *n=84* | |  | |
| Total bone content (mg/mm) | | 74.5 (73.1, 75.8) | | | 75.3 (72.7, 77.9) | | 0.578 | |
| Total bone area (mm^2^) | | 117 (115, 119) | | | 118 (114, 122) | | 0.735 | |
| Total bone density (mg/cm­^3^) | | 645 (633, 657) | | | 649 (626, 672) | | 0.781 | |
| Cortical and subcortical bone content (mg/mm) | | 64.2 (62.9, 65.4) | | | 64.9 (62.4, 67.4) | | 0.602 | |
| Cortical and subcortical bone area (mm^2^) | | 55.7 (54.7, 56.8) | | | 57.0 (55.0, 59.1) | | 0.264 | |
| Cortical and subcortical bone density (mg/cm­^3^) | | 1151 (1142, 1161) | | | 1139 (1120, 1157) | | 0.227 | |
| Polar section modulus (mm^3^) | | 78.8 (75.5, 82.1) | | | 78.0 (71.5, 84.5) | | 0.828 | |
| Polar moment of inertia (mm^4^) | | 643 (614, 672) | | | 643 (586, 700) | | 0.995 | |
| SSI_polar_ (mm^3^) | | 219 (214, 224) | | | 219 (209, 229) | | 0.929 | |
| SSI_x_ (mm^3^) | | 109 (106, 111) | | | 110 (105, 114) | | 0.745 | |
| SSI_y­_ (mm^3^) | | 147 (144, 151) | | | 148 (141, 156) | | 0.800 | |
| **Longitudinal bone measures (2-year change from baseline)** | | | | | | | | |
| *4% Radius* | *n* | |  | *n* | |  | |  |
| % ∆ Total bone area | 431 | | 0.1 (–1.1, 1.2) | 109 | | 1.7 (–0.5, 4.0) | | 0.204 |
| % ∆ Total bone density | 432 | | –2.3 (–3.3, –1.3) | 110 | | –3.7 (–5.6, –1.7) | | 0.206 |
| % ∆ Trabecular bone area | 431 | | –0.5 (–1.8, 0.9) | 109 | | 1.5 (–1.2, 4.2) | | 0.197 |
| % ∆ Trabecular bone density | 429 | | 0.5 (–0.1, 1.2) | 111 | | –0.5 (–1.7, 0.6) | | 0.114 |
| % ∆ BSI­_C_ | 433 | | –4.4 (–5.7, –2.9) | 111 | | –5.1 (–7.8, –2.7) | | 0.385 |
| *4% Tibia* |  | |  |  | |  | |  |
| % ∆ Total bone area | 478 | | –1.2 (–1.5, –0.8) | 119 | | –1.4 (–2.1, –0.7) | | 0.518 |
| % ∆ Total bone density | 476 | | –0.4 (–0.7, –0.1) | 121 | | –0.6 (–1.3, 0.0) | | 0.495 |
| % ∆ Trabecular bone area | 477 | | –1.9 (–2.3, –1.5) | 119 | | –2.0 (–2.8, –1.2) | | 0.734 |
| % ∆ Trabecular bone density | 478 | | –0.3 (–0.6, 0.1) | 121 | | –0.5 (–1.2, 0.2) | | 0.541 |
| % ∆ BSI­_C_ | 477 | | –2.0 (–2.6, –1.3) | 121 | | –3.0 (–4.4, –1.7) | | 0.187 |
| *15% Radius* |  | |  |  | |  | |  |
| % ∆ Total bone area | 292 | | 2.7 (2.0, 3.3) | 76 | | 1.5 (0.1, 2.8) | | 0.113 |
| % ∆ Total bone density | 292 | | –3.5 (–4.2, –2.9) | 77 | | –2.7 (–3.9, –1.5) | | 0.263 |
| % ∆ Cortical and subcortical bone area | 289 | | –1.0 (–2.1, 0.1) | 79 | | –1.0 (–3.1, 1.1) | | 0.981 |
| % ∆ Cortical and subcortical bone density | 291 | | **–3.1 (–4.0, –2.3)** | 78 | | **–1.2 (–2.8, 0.5)** | | **0.042** |
| % ∆ SSI_polar_ | 288 | | –1.1 (–1.8, –0.4) | 78 | | –0.9 (–2.2, 0.5) | | 0.777 |

Data are adjusted means (95%CI) for cross-sectional bone measures or adjusted percentage mean changes (95%CI) for longitudinal bone measures. Analyses adjusted for age, BMI and original treatment allocation. Analyses of longitudinal bone measures conducted on absolute change data and additionally adjusted for baseline bone data. BSI_C_ = Compressive Bone Strength Index, SSI = Stress Strain Index. Bold indicates statistical significance *p*<0.05.

**Supplementary Table 2.** Cross-sectional and longitudinal bone measures stratified by abdominal aortic calcification (AAC) extent at baseline.

|  | **AAC extent at baseline** | | | | | | |
| --- | --- | --- | --- | --- | --- | --- | --- |
|  | **Low (AAC24 = 0-1)** | | **Moderate (AAC24 = 2-5)** | | **Extensive (AAC24 ≥ 6)** | | ***p* value** |
| **Cross-sectional bone measures (baseline)** |  | |  | |  | |  |
| *4% Radius* | *n=216* | | *n=246* | | *n=160* | |  |
| Total bone content (mg/mm) | 82.3 (80.4, 84.3) | | 82.4 (80.6, 84.3) | | 81.1 (78.8, 83.5) | | 0.665 |
| Total bone area (mm^2^) | 281 (274, 288) | | 285 (278, 291) | | 277 (269, 286) | | 0.417 |
| Total bone density (mg/cm­^3^) | 300 (291, 308) | | 297 (289, 305) | | 299 (289, 309) | | 0.902 |
| Trabecular content (mg/mm) | 40.2 (38.7, 41.8) | | 40.9 (39.5, 42.4) | | 39.4 (37.7, 41.2) | | 0.436 |
| Trabecular area (mm^2^) | 217 (210, 223) | | 220 (214, 226) | | 214 (207, 222) | | 0.463 |
| Trabecular density (mg/cm­^3^) | 188 (182, 194) | | 189 (183, 195) | | 187 (179, 194) | | 0.885 |
| BSI­_C_ (g^2^/cm^4^) | 0.254 (0.242, 0.266) | | 0.253 (0.241, 0.264) | | 0.248 (0.234, 0.262) | | 0.829 |
| *4% Tibia* | *n=216* | | *n=251* | | *n=162* | |  |
| Total bone content (mg/mm) | 278 (272, 284) | | 285 (280, 290) | | 278 (271, 285) | | 0.141 |
| Total bone area (mm^2^) | 1192 (1174, 1209) | | 1203 (1186, 1219) | | 1174 (1154, 1195) | | 0.107 |
| Total bone density (mg/cm­^3^) | 235 (230, 240) | | 239 (234, 244) | | 238 (232, 244) | | 0.521 |
| Trabecular bone content (mg/mm) | 229 (223, 234) | | 235 (230, 241) | | 229 (222, 235) | | 0.152 |
| Trabecular bone area (mm^2^) | 1069 (1052, 1086) | | 1080 (1064, 1096) | | 1054 (1035, 1074) | | 0.137 |
| Trabecular bone density (mg/cm­^3^) | 215 (210, 221) | | 220 (215, 225) | | 218 (212, 225) | | 0.489 |
| BSI­_C_ (g^2^/cm^4^) | 0.669 (0.642, 0.697) | | 0.701 (0.675, 0.726) | | 0.677 (0.645, 0.709) | | 0.247 |
| *15% Radius* | *n=204* | | *n=236* | | *n=148* | |  |
| Total bone content (mg/mm) | 75.2 (73.1, 77.2) | | 74.1 (72.3, 76.0) | | 74.7 (72.3, 77.1) | | 0.760 |
| Total bone area (mm^2^) | 117 (114, 120) | | 118 (115, 120) | | 116 (112, 119) | | 0.735 |
| Total bone density (mg/cm­^3^) | 651 (633, 669) | | 636 (620, 653) | | 655 (633, 676) | | 0.324 |
| Cortical and subcortical bone content (mg/mm) | 65.0 (63.0, 66.9) | | 63.7 (62.0, 65.5) | | 64.5 (62.2, 66.7) | | 0.638 |
| Cortical and subcortical bone area (mm^2^) | 56.9 (55.3, 58.5) | | 55.5 (54.0, 56.9) | | 55.7 (53.8, 57.5) | | 0.367 |
| Cortical and subcortical bone density (mg/cm­^3^) | 1143 (1129, 1158) | | 1147 (1134, 1160) | | 1159 (1142, 1176) | | 0.357 |
| Polar section modulus (mm^3^) | 80.2 (75.1, 85.3) | | 76.6 (72.1, 81.2) | | 79.9 (74.0, 85.8) | | 0.525 |
| Polar moment of inertia (mm^4^) | 658 (613, 703) | | 628 (587, 668) | | 648 (596, 700) | | 0.600 |
| SSI_polar_ (mm^3^) | 219 (211, 226) | | 220 (213, 227) | | 219 (210, 228) | | 0.943 |
| SSI_x_ (mm^3^) | 109 (106, 113) | | 108 (105, 112) | | 109 (104, 113) | | 0.924 |
| SSI_y­_ (mm^3^) | 148 (142, 153) | | 148 (143, 153) | | 147 (140, 154) | | 0.970 |
| **Longitudinal bone measures (2-year change from baseline)** | | | | | | | |
| *4% Radius* | *n* |  | *n* |  | n |  |  |
| % ∆ Total bone area | 191 | 0.3 (–1.4, 2.1) | 213 | 0.4 (–1.3, 2.0) | 136 | 0.6 (–1.5, 2.7) | 0.975 |
| % ∆ Total bone density | 191 | –2.7 (–4.2, –1.2) | 212 | –1.5 (–2.9, 0.0) | 139 | –4.2 (–6.0, –2.3) | 0.081 |
| % ∆ Trabecular bone area | 191 | –0.2 (–2.2, 1.9) | 213 | –0.3 (–2.2, 1.6) | 136 | 0.4 (–2.1, 2.8) | 0.905 |
| % ∆ Trabecular bone density | 192 | –0.5 (–1.4, 0.4) | 210 | 0.9 (0.0, 1.8) | 138 | 0.6 (–0.5, 1.7) | 0.076 |
| % ∆ BSI­_C_ | 193 | –5.4 (–7.4, –3.4) | 212 | –2.7 (–4.6, –0.6) | 139 | –6.2 (–8.9, –3.7) | 0.053 |
| *4% Tibia* |  |  |  |  |  |  |  |
| % ∆ Total bone area | 203 | –1.6 (–2.1, –1.0) | 241 | –0.8 (–1.3, –0.3) | 153 | –1.3 (–1.9, –0.6) | 0.132 |
| % ∆ Total bone density | 205 | –0.4 (–0.9, 0.0) | 239 | –0.4 (–0.8, 0.1) | 153 | –0.6 (–1.1, –0.1) | 0.784 |
| % ∆ Trabecular bone area | 203 | –2.2 (–2.8, –1.6) | 240 | –1.5 (–2.1, –1.0) | 153 | –2.1 (–2.8, –1.4) | 0.241 |
| % ∆ Trabecular bone density | 205 | –0.3 (–0.8, 0.2) | 240 | –0.2 (–0.7, 0.2) | 154 | –0.5 (–1.1, 0.1) | 0.698 |
| % ∆ BSI­_C_ | 205 | –2.7 (–3.7, –1.6) | 240 | –1.6 (–2.5, –0.6) | 153 | –2.5 (–3.8, –1.4) | 0.301 |
| *15% Radius* |  |  |  |  |  |  |  |
| % ∆ Total bone area | 124 | 2.1 (1.0, 3.2) | 152 | 2.5 (1.5, 3.4) | 92 | 2.8 (1.5, 4.0) | 0.708 |
| % ∆ Total bone density | 125 | –3.2 (–4.2, –2.2) | 153 | –3.4 (–4.3, –2.5) | 91 | –3.6 (–4.7, –2.4) | 0.915 |
| % ∆ Cortical and subcortical bone area | 127 | –0.8 (–2.5, 0.8) | 152 | –1.7 (–3.2, –0.2) | 89 | –0.1 (–2.2, 2.0) | 0.453 |
| % ∆ Cortical and subcortical bone density | 126 | –1.7 (–3.0, –0.4) | 153 | –3.6 (–4.8, –2.4) | 90 | –2.6 (–4.2, –1.1) | 0.112 |
| % ∆ SSI_polar_ | 125 | –0.6 (–1.7, 0.5) | 151 | –0.9 (–1.9, 0.1) | 90 | –1.8 (–3.1, –0.5) | 0.414 |

Data are adjusted means (95%CI) for cross-sectional bone measures or adjusted percentage mean changes (95%CI) for longitudinal bone measures. Analyses adjusted for age, BMI and original treatment allocation. Analyses of longitudinal bone measures conducted on absolute change data and additionally adjusted for baseline bone data. BSI_C_ = Compressive Bone Strength Index, SSI = Stress Strain Index.

**Supplementary Table 3.** Cross-sectional and longitudinal bone measures stratified by progression of abdominal aortic calcification (AAC) from in the 4-5 years prior to baseline.

|  | | **AAC progression in the 4-5 years prior to baseline** | | | | | | |  |
| --- | --- | --- | --- | --- | --- | --- | --- | --- | --- |
|  | | **Yes (∆ AAC24 ≥ 2)** | | | **No (∆ AAC24 ≤1)** | | ***p* value** | |  |
| **Cross-sectional bone measures (baseline)** | |  | | |  | |  | |  |
| *4% Radius* | | *n=154* | | | *n=376* | |  | |  |
| Total bone content (mg/mm) | | 82.3 (79.9, 84.7) | | | 82.1 (80.6, 83.6) | | 0.889 | |  |
| Total bone area (mm^2^) | | 278 (270, 287) | | | 283 (278, 289) | | 0.346 | |  |
| Total bone density (mg/cm­^3^) | | 304 (294, 314) | | | 296 (290, 302) | | 0.200 | |  |
| Trabecular content (mg/mm) | | 40.8 (39.0, 42.6) | | | 40.4 (39.2, 41.6) | | 0.710 | |  |
| Trabecular area (mm^2^) | | 215 (207, 223) | | | 219 (214, 224) | | 0.361 | |  |
| Trabecular density (mg/cm­^3^) | | 193 (186, 201) | | | 187 (182, 191) | | 0.136 | |  |
| BSI­_C_ (g^2^/cm^4^) | | 0.255 (0.241, 0.269) | | | 0.250 (0.241, 0.259) | | 0.581 | |  |
| *4% Tibia* | | *n=159* | | | *n=375* | |  | |  |
| Total bone content (mg/mm) | | 284 (277, 291) | | | 281 (276, 285) | | 0.448 | |  |
| Total bone area (mm^2^) | | 1183 (1163, 1203) | | | 1198 (1185, 1211) | | 0.213 | |  |
| Total bone density (mg/cm­^3^) | | 242 (235, 248) | | | 236 (232, 240) | | 0.141 | |  |
| Trabecular bone content (mg/mm) | | 235 (228, 241) | | | 231 (227, 236) | | 0.404 | |  |
| Trabecular bone area (mm^2^) | | 1062 (1043, 1081) | | | 1075 (1063, 1088) | | 0.263 | |  |
| Trabecular bone density (mg/cm­^3^) | | 222 (216, 229) | | | 217 (212, 221) | | 0.141 | |  |
| BSI­_C_ (g^2^/cm^4^) | | 0.703 (0.670, 0.735) | | | 0.679 (0.658, 0.700) | | 0.234 | |  |
| *15% Radius* | | *n=104* | | | *n=247* | |  | |  |
| Total bone content (mg/mm) | | 73.4 (71.0, 75.8) | | | 75.3 (73.8, 76.9) | | 0.192 | |  |
| Total bone area (mm^2^) | | 115 (111, 118) | | | 118 (116, 121) | | 0.098 | |  |
| Total bone density (mg/cm­^3^) | | 647 (627, 668) | | | 643 (630, 656) | | 0.737 | |  |
| Cortical and subcortical bone content (mg/mm) | | 63.4 (61.1, 65.6) | | | 65.0 (63.6, 66.5) | | 0.228 | |  |
| Cortical and subcortical bone area (mm^2^) | | 55.0 (53.1, 56.8) | | | 56.8 (55.6, 58.0) | | 0.102 | |  |
| Cortical and subcortical bone density (mg/cm­^3^) | | 1155 (1138, 1172) | | | 1145 (1134, 1156) | | 0.301 | |  |
| Polar section modulus (mm^3^) | | 77.5 (71.6, 83.5) | | | 79.6 (75.7, 83.4) | | 0.570 | |  |
| Polar moment of inertia (mm^4^) | | 626 (573, 679) | | | 656 (622, 690) | | 0.355 | |  |
| SSI_polar_ (mm^3^) | | 215 (206, 224) | | | 222 (217, 228) | | 0.174 | |  |
| SSI_x_ (mm^3^) | | 106 (101, 110) | | | 111 (108, 114) | | 0.058 | |  |
| SSI_y­_ (mm^3^) | | 146 (139, 152) | | | 149 (145, 154) | | 0.375 | |  |
| **Longitudinal bone measures (2-year change from baseline)** | | | | | | | | |  |
| *4% Radius* | *n* | |  | *n* | |  | |  | |
| % ∆ Total bone area | 134 | | 0.4 (–1.6, 2.5) | 328 | | 0.4 (–0.9, 1.7) | | 0.971 | |
| % ∆ Total bone density | 135 | | –1.6 (–3.3, 0.2) | 327 | | –2.7 (–3.8, –1.6) | | 0.289 | |
| % ∆ Trabecular bone area | 134 | | –0.1 (–2.5, 2.3) | 328 | | –0.1 (–1.6, 1.5) | | 0.973 | |
| % ∆ Trabecular bone density | 133 | | 0.7 (–0.4, 1.7) | 328 | | –0.1 (–0.7, 0.6) | | 0.245 | |
| % ∆ BSI­_C_ | 135 | | –2.8 (–5.4, –0.4) | 329 | | –5.1 (–6.5, –3.4) | | 0.164 | |
| *4% Tibia* |  | |  |  | |  | |  | |
| % ∆ Total bone area | 150 | | –0.9 (–1.5, –0.2) | 360 | | –1.4 (–1.9, –1.0) | | 0.140 | |
| % ∆ Total bone density | 149 | | –0.4 (–1.0, 0.1) | 359 | | –0.4 (–0.7, 0.0) | | 0.846 | |
| % ∆ Trabecular bone area | 149 | | –1.7 (–2.4, –1.0) | 360 | | –2.1 (–2.5, –1.6) | | 0.374 | |
| % ∆ Trabecular bone density | 150 | | –0.2 (–0.8, 0.4) | 360 | | –0.3 (–0.7, 0.1) | | 0.888 | |
| % ∆ BSI­_C_ | 149 | | –1.7 (–2.9, –0.5) | 360 | | –2.3 (–3.1, –1.5) | | 0.423 | |
| *15% Radius* |  | |  |  | |  | |  | |
| % ∆ Total bone area | 92 | | 3.3 (2.0, 4.5) | 227 | | 2.1 (1.3, 2.8) | | 0.129 | |
| % ∆ Total bone density | 92 | | –3.6 (–4.8, –2.5) | 227 | | –3.4 (–4.1, –2.7) | | 0.805 | |
| % ∆ Cortical and subcortical bone area | 89 | | –0.7 (–2.8, 1.5) | 229 | | –1.3 (–2.6, –0.1) | | 0.583 | |
| % ∆ Cortical and subcortical bone density | 91 | | –3.9 (–5.5, –2.4) | 228 | | –2.3 (–3.3, –1.4) | | 0.077 | |
| % ∆ SSI_polar_ | 92 | | –1.2 (–2.5, 0.1) | 224 | | –1.0 (–1.8, –0.2) | | 0.831 | |

Data are adjusted means (95%CI) for cross-sectional bone measures or adjusted percentage mean changes (95%CI) for longitudinal bone measures. Analyses adjusted for age, BMI, original treatment allocation and initial AAC24. Analyses of longitudinal bone measures conducted on absolute change data and additionally adjusted for baseline bone data. BSI_C_ = Compressive Bone Strength Index, SSI = Stress Strain Index.
